# Statistical issues in the analysis of Illumina data

**DOI:** 10.1186/1471-2105-9-85

**Published:** 2008-02-06

**Authors:** Mark J Dunning, Nuno L Barbosa-Morais, Andy G Lynch, Simon Tavaré, Matthew E Ritchie

**Affiliations:** 1 Department of Oncology, University of Cambridge, CRUK Cambridge Research Institute, Li Ka Shing Centre, Robinson Way, Cambridge CB2 0RE, UK

## Abstract

**Background:**

Illumina bead-based arrays are becoming increasingly popular due to their high degree of replication and reported high data quality. However, little attention has been paid to the pre-processing of Illumina data. In this paper, we present our experience of analysing the raw data from an Illumina spike-in experiment and offer guidelines for those wishing to analyse expression data or develop new methodologies for this technology.

**Results:**

We find that the local background estimated by Illumina is consistently low, and subtracting this background is beneficial for detecting differential expression (DE). Illumina's summary method performs well at removing outliers, producing estimates which are less biased and are less variable than other robust summary methods. However, quality assessment on summarised data may miss spatial artefacts present in the raw data. Also, we find that the background normalisation method used in Illumina's proprietary software (BeadStudio) can cause problems with a standard DE analysis. We demonstrate that variances calculated from the raw data can be used as inverse weights in the DE analysis to improve power. Finally, variability in both expression levels and DE statistics can be attributed to differences in probe composition. These differences are not accounted for by current analysis methods and require further investigation.

**Conclusion:**

Analysing Illumina expression data using BeadStudio is reasonable because of the conservative estimates of summary values produced by the software. Improvements can however be made by not using background normalisation. Access to the raw data allows for a more detailed quality assessment and flexible analyses. In the case of a gene expression study, data can be analysed on an appropriate scale using established tools. Similar improvements can be expected for other Illumina assays.

## Background

A BeadArray is an array of randomly positioned, three micron diameter, silica beads. A specific oligonucleotide sequence is assigned to each *bead type*, which is replicated about 30 times on an array. A series of decoding hybridisations is used to identify every bead [1]. BeadArrays can be used in a wide range of genomic applications including SNP genotyping, methylation profiling, and copy number variation analysis. In this paper we concentrate on data from single-channel gene expression BeadArrays [2]. After hybridisation and washing, each array is scanned by Illumina's scanning software (BeadScan) to produce an image in the Tagged Image File Format (TIFF), along with files in a proprietary file format containing intensity and location information. The latest version of BeadScan can also output a text file giving the identity and position of each bead on the array. The collection of TIFF images and text files is referred to as the *bead level data *for an experiment and are presented in the same format, regardless of the assay used.

Analysis of BeadArray data is routinely carried out using the BeadStudio application developed by Illumina. This software takes the proprietary files and produces *bead summary data*, comprising a mean and standard error on the original unlogged scale, for each bead type on each array. When the raw data are analysed using BeadStudio, there is no control over image processing or the method used to combine the replicate observations for a given bead type into a single, summarised, value. Also, the user loses the ability to perform a detailed quality assessment of the data.

Information about how to obtain bead level data has only recently been released and these data cannot be generated retrospectively. Therefore, no publications have taken the processing of bead level data into account apart from our own preliminary investigations [3]. In comparison to Affymetrix, which is an established technology, there is a lack of in-depth literature on the low-level analysis of Illumina data. Publications involving Illumina expression data tend to use Illumina's recommendations for normalisation, with the exception of [4] who found this method to have a negative impact on data quality and who used quantile normalisation instead. Also, there is no publicly available dataset for which the bead level data may be obtained and for which there is some expectation about the results. Such datasets are available for Affymetrix and have allowed researchers to understand more about the technology and to develop new analysis methods [5].

This paper presents our guidelines regarding the pre-processing of Illumina data and explores some common issues raised during the analysis of other microarray platforms. After discussing the bead level properties and quality assessment of a spike-in experiment, we investigate the effects of the image processing and summarisation methods used by Illumina on a typical DE analysis.

Image processing is an important consideration for microarrays and involves calculating foreground intensities using the pixels that make up each feature on the array, and estimating a local background intensity using the pixels surrounding each feature. A background correction calculation is then used to correct for non-specific or random contributions to the overall signal. Within BeadScan, the local background measures are subtracted from the bead foreground values to produce the intensities in the bead level text files. This method is the same for all Illumina technologies. Previous studies have shown that background subtraction introduces missing values, increases variability and has a negative impact on the detection of differentially expressed genes [6,7]. Therefore, it is crucial to investigate the automatic background adjustment used within BeadScan.

In addition to local background subtraction, Illumina also recommends a secondary calibration procedure for expression data, known as *background normalisation*, which is performed on summarised data. For a given array, this method involves subtracting the mean intensity of the negative controls (bead types with randomly permuted probe sequences attached and no targets in the genome) from each bead summary value. This is intended to compensate for differences between arrays in both non-specific binding of dye and cross-hybridisation. After background normalisation, an additional normalisation using rank invariant genes or cubic splines can be carried out between arrays. We demonstrate the effect of applying this normalisation to the spike-in experiment.

For Affymetrix data, it has been shown that the base composition of the 25-mer probes can have an effect on the observed intensity, and methods have been proposed to deal with this effect [8]. Other probe effects have also been described for two-colour microarrays with longer probes [9], but have not been reported for Illumina data, which typically uses 50-mer probes. Additionally, the reliability of Affymetrix probe sets has been called into question, with a large percentage of probes on an array sometimes not mapping to the intended transcript. Naturally, this can lead to misleading conclusions in a DE study [10]. We investigate if such effects are apparent in Illumina gene expression data.

### Data

The Illumina spike-in experiment investigated in this paper consists of eight customised Mouse-6 version 1 BeadChips hybridised with a complex mouse background. In addition to the ~48,000 bead types included as standard, the bead pool for these chips was modified to include 33 bead types chosen to target 9 different bacterial and viral genes absent from the Mouse genome [2]. These 33 bead types are referred to as *spikes *in this paper and the remaining bead types on the array are referred to as *non-spikes*. Each array also includes a number of standard Illumina controls, including 1616 negative controls. Each BeadChip comprises six arrays and each array is made up of two strips on the chip surface. The first strip interrogates targets from the curated MEEBO database, and the second strip mostly contains targets from the RefSeq and RIKEN databases.

The spikes were added at concentrations of 1000, 300, 100, 30, 10 and 3 pM on the six arrays from the first four BeadChips. A further four chips were hybridised with spikes at concentrations of 1, 0.3, 0.1, 0.03, 0.01 and 0 pM. The spikes on a given array were all added at the same concentration. Each concentration was allocated to the same position on all replicate BeadChips. For example, 1000 pM was always array 1 on a chip and 300 pM was array 2 and so on. The spike-in experiment was designed and scanned by Illumina. Raw data from the experiment, which includes the TIFF image and text file for each strip, are available as supplementary materials [11]. Annotation information (including the 50-mer probe sequence attached to each bead type), additional figures and R scripts are also available online [11].

## Results

### Bead level issues

**Figure 1:**
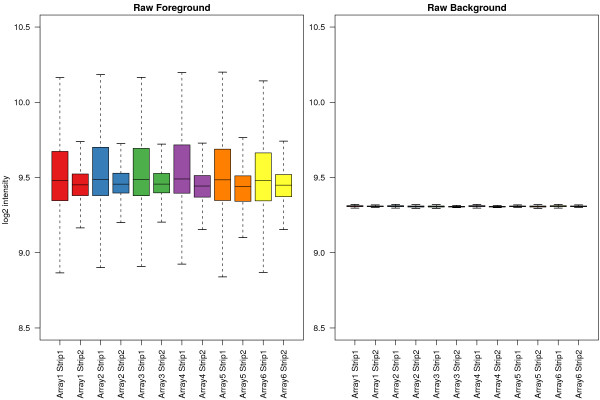
Raw foreground and background intensities for each strip on a typical BeadChip. Each BeadArray is made up of two strips (colour-coded) on the chip surface. The consistency of foreground and background signals between arrays is evident from this plot, as is the tendency for beads from the first strip to have higher intensities than those from the second strip, which is related to chip design.

Figure 1 shows the raw foreground intensities and local background estimates for all beads on each strip from a typical BeadChip. We see that the signal on the first strip is generally higher and has a greater dynamic range than the signal from the second strip. The local background estimates show very low variability both within and between arrays, with a median of 634 on the original scale (9.3 on the log_2 _scale). The distribution of background signal is the same for all strips, despite differences in foreground signal. Closer inspection of the raw images revealed that although the higher intensity pixels within the same size window varied between bead centres, the median value obtained within the local background of a bead is very similar to the overall median of background pixels on the array and not much higher than the five lowest pixels, which are used in the calculation of local background. When looking at the replicates of a particular bead type, the variability of the foreground estimates was substantially larger than that of the background estimates (data not shown).

Access to the bead level data allowed us to identify a significant spatial artefact on one BeadChip in the experiment. An error in the scanning of the chip resulted in the x coordinates of many beads on the left-hand side taking negative values. Consequently, the Illumina algorithm for image processing was unable to calculate foreground and background intensities for these beads and set their foreground intensities to zero. This problem affected between 4.4% and 7.3% of the total number of beads from each strip on this chip, with the percentage of affected beads decreasing from the top to the bottom of the chip. These beads were subsequently removed as outliers by Illumina's summary algorithm. No other significant spatial effects were found on the remaining chips and arrays. We note that after background correcting the bead level data, the median percentage of beads with negative intensities on a strip was 0.32% with a 75th percentile of 0.56%.

**Figure 2:**
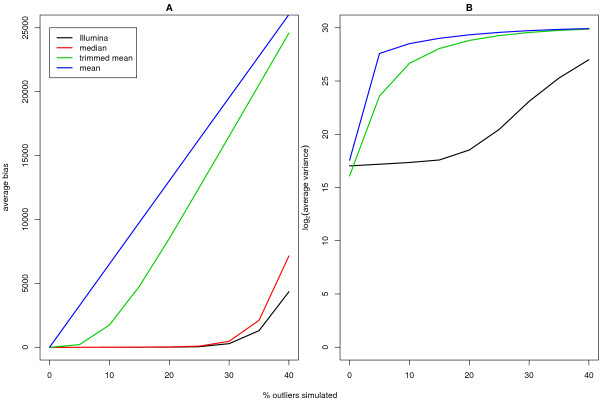
The average bias (A) and log_2 _variance (B) versus percentage of simulated outliers plotted for each summary method. In panel A, we see that Illumina's summary method can handle up to about 20% of saturated intensities before the bias starts to increase dramatically. The trimmed mean breaks down much earlier, at around 5%. The median is comparable to Illumina's method. Similar trends can be noticed in the variance (B).

Before proceeding with an analysis of summarised data, we investigated how robust Illumina's technology is to the spatial effects observed above. Arrays with varying numbers of saturated beads were simulated as described in Methods. These data sets aimed at assessing how many outliers could be tolerated by Illumina'a default summary method compared to other methods (mean, trimmed mean and median). Figure 2 shows the average bias and variance versus the percentage of outliers introduced. Illumina's summary method performs best overall, with the lowest bias (Figure 2A) and variance (Figure 2B). After around 20% of the bead intensities become saturated, the bias and variance start to increase. Using a trimmed mean which excludes 10% of the smallest and largest intensities, we see an increase in bias and variance after more than 5% of the beads are saturated. This is not surprising as the outliers are not simulated to be symmetric around the mean, which this method is suited to handle. The median offers similar robustness to Illumina's method. Similar results were obtained if the analysis was performed on the log-scale, or if the data were censored at 0 rather than at 2^16 ^(refer to supplementary materials).

After applying the Illumina summary method, the median number of beads per bead type on an array was 36 with 25th and 75th percentiles of 31 and 41 respectively. The median number of beads per bead type per array removed as outliers was 1 with a 95th percentile of 4.

### The effects of pre-processing on DE analysis

**Figure 3:**
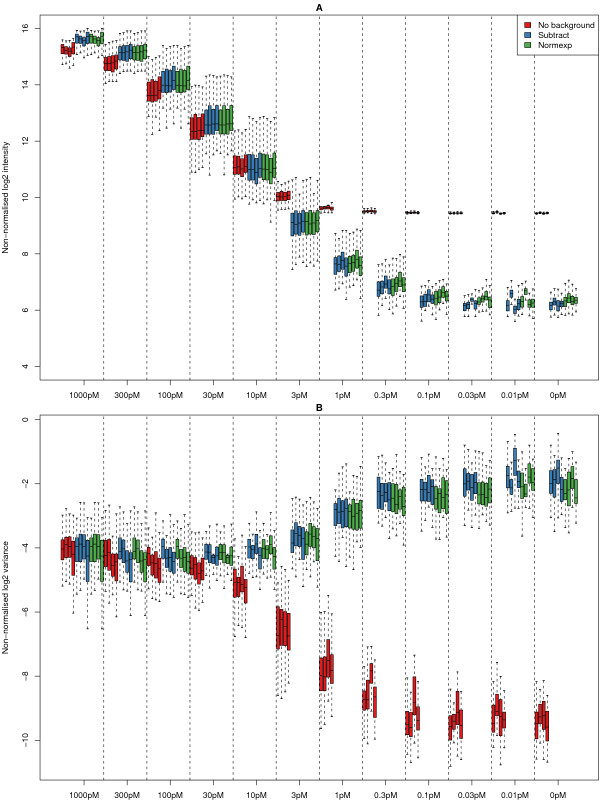
Boxplots of the means (A) and variances (B) for the 33 spikes on all arrays in the experiment after outlier removal. The boxplots are arranged in decreasing order of spike concentration, with different background correction methods labeled in different colours. The no background adjustment option shows dramatic attenuation in signal, which begins at a higher concentration than the other background correction options.

In Figure 3 we show the bead type means and variances calculated on the log_2 _scale, for the 33 spikes across all arrays in the experiment using three background correction methods (no background adjustment, background subtraction and normexp model-based adjustment – see Methods). These boxplots of non-normalised data are arranged according to the concentration of the spikes on the array. Different background correction methods are shown in different colours and data from each array are plotted in a separate boxplot. Given the design of the experiment, we would expect to see a decrease in observed intensity as the concentration of the spikes decreases.

Note that even though the data shown are not normalised, we can see that the replicates of the same array processed using the same method show low variability and only slight differences in the medians. The same trend can be seen for all background correction methods. A saturation effect can to be seen between 300 pM and 1000 pM, as the increase in the concentration of the spikes is not reflected in a change in observed intensity. At 3 pM, there is a clear difference between arrays with no background adjustment and arrays where a local background estimate has been subtracted. The linear relationship between spike concentration and observed intensity persists below 3 pM for the background subtracted data, whereas without background adjustment, an attenuation in signal is evident below 3 pM.

The variances of each method are similar in the range 1000 pM to 100 pM. However, at 10 pM we see a steep decrease in the variability for the non-adjusted data, whilst the background subtracted data shows a slight increase. The rate of decrease in variability for the no background adjustment option is greater than the rate of increase in variability for the subtracted data. For concentrations of 0.1 pM and below, the variance of the spikes does not decrease any further with decreasing concentration.

**Figure 4:**
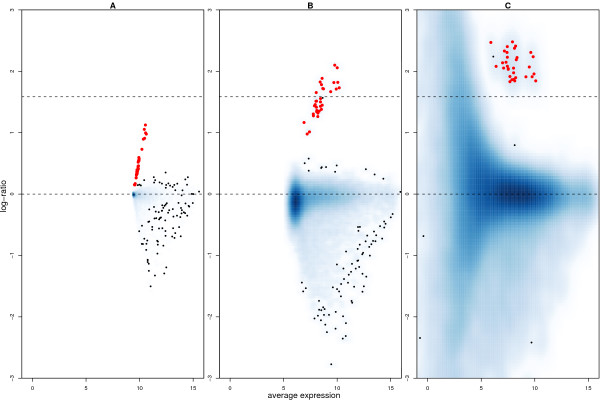
MA-plots comparing the bead summary values for one array with spike concentration 3 pM to an array with spike concentration 1 pM. An increased density of points is indicated by darker shades of blue. Red points highlight the spikes. The horizontal line at *M *= 1.73 represents the expected log-ratio for the spikes, and the line at *M *= 0 is the desired level for the remaining non-spikes. Each panel shows the data processed using different background correction methods. Panel A shows the data with no background adjustment, while in panel B local background has been subtracted and in panel C the data has been background subtracted and background normalised. When the data are background subtracted, the range of *M *and *A*-values increases and the spikes are closer to the true value than for the non-adjusted data. Background normalistion produces the most variable *M*-values and over-estimates the *M*-values for the spikes.

We now quantify how well the expected change in spike concentration is recovered by different background correction methods. In Figure 4 we show an MA-plot (see Methods) of the log_2 _transformed data from an array with spikes at 3 pM and an array with spikes at 1 pM. The data shown in Figure 4A were not background adjusted and we can see that the range of *M*-values is very low for all genes. The largest *M*-value we see is around 1.2 and the *A*-values are in the range 10 to 15. The observed log-ratios for the spikes are much lower than the expected value of 1.73. Figure 4B shows the same data after background subtraction. We see a wider range of *M *and *A *values compared to Figure 4A, and the log-ratios for the spikes are closer to the expected value on average. Notice that although these data have not been normalised, the non-spikes lie around *M *= 0, indicating no differential expression.

In Figure 4C we show the same comparison for data which have been background subtracted and background normalised. This is equivalent to processing the raw data using BeadStudio's recommended settings. For visualisation purposes, and to compare with the other methods, we log_2 _transformed the background normalised data. The difference that this makes to the MA-plot is striking. We see a much increased range of *M*-values as *A *decreases. There are clearly a large number of genes which would be selected as differentially expressed if a simple cut-off approach were used, even though few genes are expected to change for this comparison. In addition, the log-ratios of the spikes are systematically over-estimated by this method. There are also a large number of bead types with negative intensities on each array after applying background normalisation, ranging from 11.18% to 49.35% (median 39.06%). These become missing values after a log_2 _transformation, which is undesirable in downstream analyses.

**Figure 5:**
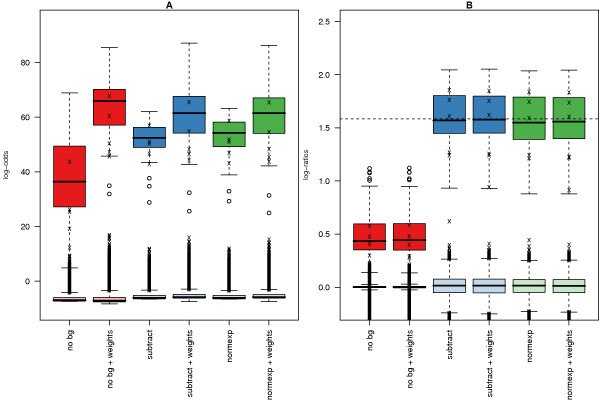
Boxplots of the log-odds scores (A) and log-ratios (B) obtained after fitting a linear model to all genes across all arrays in the spike experiment and making contrasts between 3 pM and 1 pM. A separate box is shown for each background correction method with and without bead variances as inverse weights in the linear model. Two separate boxplots are shown for each method and weighting scheme to indicate the log-odds scores for the spikes (bold colours) and non-spikes (transparent colours). The use of weights improves the log-odds scores for the spikes without increasing the log-odds for the non-spikes, which represents an increase in power to detect true DE. In panel B, we show that the log-ratios for the spikes are under-estimated when the data is not background adjusted, whereas the background subtracted and normexp processed data recover values much closer to the true log fold-change (dashed line, *M *= 1.73).

Analyses of replicated arrays use statistical methods to determine genes that show DE for a particular contrast of interest. In this paper, we chose the linear model approach of [12]. Figure 5A shows the log-odds scores for the contrast between 3 pM and 1 pM and for data processed using different background correction methods. Separate boxplots are shown for the spikes and non-spikes (solid and transparent colour respectively) and results are shown both with and without weights in the model (see Methods). Outliers for the non-spikes are indicated by crosses. Background subtraction is seen to increase the log-odds of being differentially expressed for the spikes. Moreover, a greater distinction between the log-odds of the spikes and non-spikes is seen after background subtraction.

Five non-spikes are seen to have high log-odds both before and after weights were used. These bead types were ranked amongst the spikes for all contrasts and had a similar expression profile to the spikes. Further investigation revealed these probes are controls from the MEEBO database and not used in current Illumina chips. Generally, the spikes were the top ranked probes for each contrast with very few false discoveries. The choice of background correction method was found to have little impact on the number of false discoveries (see supplementary materials).

When weights are used in the linear model for a particular correction method, we see an increase in the log-odds scores calculated for the spikes. At the same time, we do not see a substantial change for the non-spikes. The most dramatic increase in log-odds by using weights is seen for the non-adjusted data. It is interesting to note that the log-odds of the different methods are more comparable when weights are used. We produced the same plot for all contrasts in the linear model (data not shown). The log-odds typically increased for all contrasts in the middle of the concentration range when weights were used. However, for contrasts comparing 0.3 pM to lower concentrations, we found little improvement, or sometimes a decrease in log-odds. Figure 5B shows the estimated coefficients for the comparison between 3 pM and 1 pM. For this contrast, we would expect the spikes to have a log-ratio of log_2_(3) or 1.73. For data processed without background adjustment, the highest log-ratio seen for the spikes is not much greater than 1. For the subtract and normexp methods, the log-ratios are centered around the expected value. For other contrasts (data not shown), the log-ratios were often underestimated by all methods, especially at high and low concentrations. Pairwise contrasts 30 pM to 10 pM, 10 pM to 3 pM and 3 pM to 1 pM accurately recovered the predicted log fold-changes. The non-adjusted data consistently produced the most biased values for all contrasts.

### Probe properties and annotation considerations

**Figure 6:**
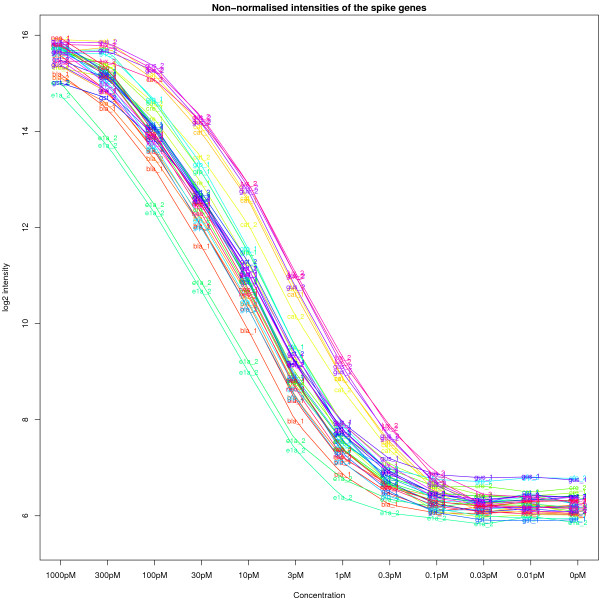
The log_2 _intensities for the 33 spikes on each array estimated using the linear model. Each spike is indicated by a different colour and line. Despite being added at the same concentration, consistent differences are seen between the spikes, for example, *ela_2 *consistently has the lowest intensity.

We now repeat a similar analysis to Figure 3, but consider the behaviour of each spike separately. In Figure 6, we show the coefficients for each spike estimated by fitting the linear model described in Methods to the background subtracted data. For clarity, each spike was labelled and coloured according to its target gene. A smoothed curve was fitted to the coefficients for each spike. Note that bead types with the same target name (e.g. *ela_2*) have the same probe sequence attached, but are located on different strips. We can clearly see different intensities for spikes at the same concentration. These differences are consistent across the concentration series. For example, *ela_2 *always shows the lowest intensity at all concentrations, whereas *gus_2 *and *lux_2 *tend to have the highest intensity. The difference between the spikes is quite dramatic for some concentrations. For instance, at 30 pM the highest intensity spikes measured are 14, whereas the lowest intensities are at 11. It is also apparent that the spikes respond differently to the decrease in concentration. The *ela_2 *spikes show a larger decrease between 1000 pM and 300 pM than the other spikes and the curve for these spikes flattens out at a higher concentration. Conversely, the spikes for *gus_2 *are flatter for concentrations 1000 pM to 100 pM, but attenuate at lower concentrations than the other spikes. Some small differences can be seen for bead types having the same probes sequence, but hybridised to different strips.

After re-annotation of the available probe sequences, bead types were categorised according to where they map in the genome. Some probe sequences were found to match to intronic and intergenic regions. The percentages of bead types on the second strip with intronic and intergenic matches were 27.67% and 9.64%, respectively, compared to 2.84% and 0.32% on the first strip. There were 16,332 and 7,317 unique genes represented on the first and second strips respectively, in addition to the 2,263 genes interrogated on both strips. As expected, re-annotation of the spikes and negative controls produced no matches in the genome.

**Figure 7:**
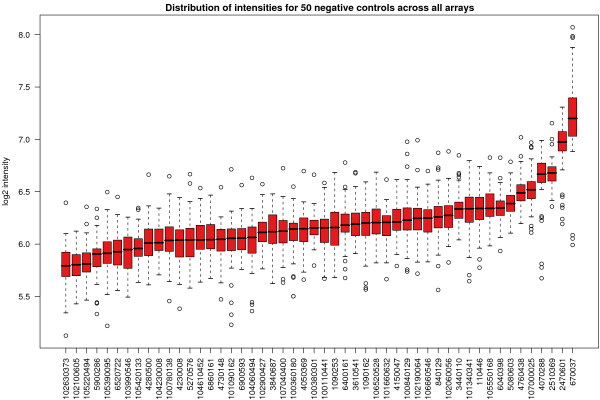
The distribution of background subtracted and summarised intensities for 50 negative controls across all arrays in the experiment, ordered by increasing median. Each control is a bead type with a random sequence attached which should not hybridise to any target in the genome. Despite this, some controls clearly appear to show consistently higher intensities than others.

An important subset of the non-spikes are the negative controls, which in addition to not changing between arrays, should have a low intensity on every array. In Figure 7, for 50 negative controls picked at random, we plot the (background adjusted) averaged values for each control over all arrays. As expected, each control shows intensities at the lower end of the values observed on an array (Figure 1). However, we do see some variation in median intensity between the different probes, with a greater than two-fold difference in intensity measured between the bead type on the far left and far right of the plot.

**Figure 8:**
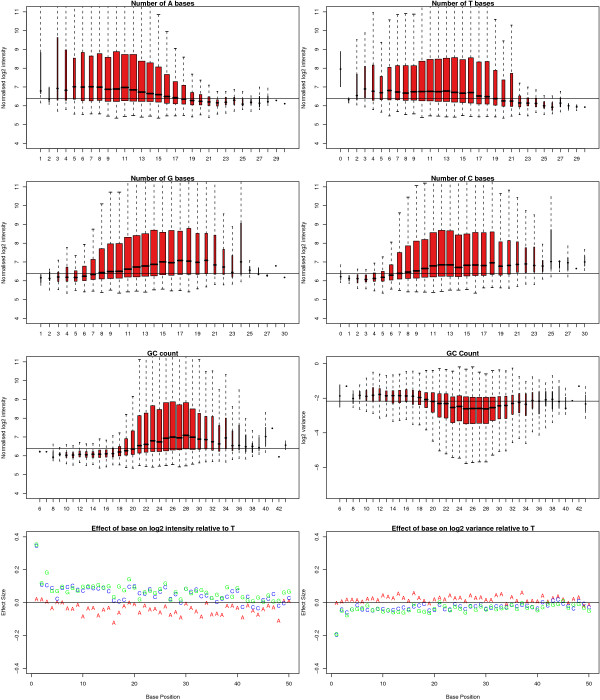
Normalised log_2 _intensities for all non-spikes on strip 1 of a particular array in the experiment grouped according to the number of As, Ts, Gs or Cs in the probe sequence. The normalised log_2 _intensities and bead type variances are also shown in terms of GC content. The width of each box is proportional to the number of observations. Probes with higher GC content are shown to have higher intensity on average and a lower variance. Finally, estimated effect sizes are shown for each base position relative to having a T at that position. The normalised intensities are seen to be higher if a G or C is present at the first base in the sequence and have a lower variance. However, no other systematic trend is seen.

In Figure 8 we see the normalised intensity of all non-spikes on strip 1 of a particular array grouped according to how many A, C, T or G bases are found in the sequence attached to each bead type. Generally, we see that an increase in the number of As or Ts in the sequence is associated with a decrease in mean intensity, whereas an increase in the number of Cs or Gs results in an increase in mean intensity. Moreover, as the GC content increases, the variance of the bead types decreases. We also see that probes with either a G or C as the first base have a higher normalised intensity and lower variance relative to having a T at that position. The distribution of GC content for the spikes was skewed towards higher GC content and showed little variation. Therefore, we did not have sufficient information to conclude a GC-related effect for these probes alone (data not shown). Similarly, we could find no evidence for an effect of the GC content on the intensity of the negative controls.

**Figure 9:**
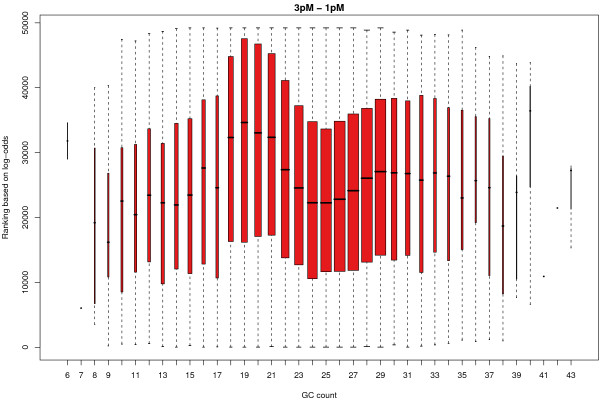
The log-odds ranking of all non-spikes on strip 1 for the contrast between 3 pM and 1 pM aggregated according to the GC content of each probe. Probes with a GC content of 18–23 are generally ranked higher in the list. The width of each box is proportional to the number of observations.

Ideally, we would like such probe effects to be removed when comparisons are made between arrays. In Figure 9 we show the ranking of the log-odds scores of the contrast shown in Figure 7 for all non-spikes on strip 1. Clearly there is a preference for bead types with 18 to 21 GCs in the sequence to be higher in the list. On average, sequences with 19 GCs are 10,000 places higher in the list than sequences with 24 GCs.

The "hump" seen in Figure 9 was evident for most contrasts in the linear model. We have observed similar trends in experiments which use Human version 1 BeadChips (data not shown).

**Figure 10:**
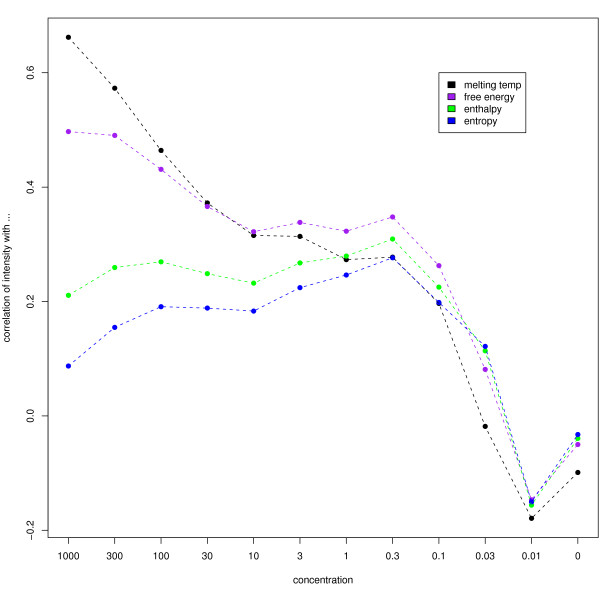
The correlation of observed spike intensity with the thermodynamic properties calculated using the spike sequences. Consistent with findings from an Affymetrix spike-in experiment, the observed spike intensities are seen to correlate well with melting temperature and free energy at high spike concentrations.

For the Affymetrix spike-in experiments, the thermodynamic properties were shown to explain some of the variation between observed intensities for different probes spiked in at a nominally consistent level [13]. In this Illumina experiment, similar strong correlations are seen at high concentrations (see Figure 10). The correlation of the negative control intensities to Δ*H*, Δ*S *and Δ*G *(see Methods) have inter-quartile ranges of 0.13 to 0.15, 0.13 to 0.16 and 0.07 to 0.09 respectively. The intensities of the negative controls are highly correlated from array to array, although the agreement between arrays is quite poor. Thus, for the most robust analysis we compare the thermodynamic properties to the average within-array rank of the negative controls. The correlations are 0.13 for Δ*S*, 0.13 for Δ*H *and 0.08 for Δ*G*. Whilst these values are small in magnitude, they are significantly non-zero.

## Discussion

### Data quality

The data produced using Illumina technology are widely reported to be of high quality. Naturally, we would still recommend careful quality assessment of Illumina arrays and not to take high data quality for granted. Whilst initial quality assessment using the raw data showed little variation between arrays, we were able to detect a consistent spatial effect on a particular BeadChip. However, we found that in this case, there was no impact on further analysis due to the random placement of beads and robust summary method used by Illumina. Although BeadStudio is capable of giving a good overview of an experiment, it will miss important artefacts on arrays, as spatial information is lost when the data are summarised. We found that the two strips for each array show consistently different intensities with the first strip showing a wider range of expression values. We have noticed this effect for other mouse expression data and also for Human-6 Version 1 chips (data not shown). The second strip contains a large number of bead types with sequences that target rare transcripts, or match to intronic or intergenic regions, which could partly explain the lower signal on this strip. The default options within the BeadStudio software combine the two strips for every array on a whole genome BeadChip. In the Version 2 whole genome BeadChips, the replicates of each bead type are spread between the two strips with potentially 20 replicates on each strip. Clearly, the summary value could be affected by any differences in underlying intensity between the strips, in which case analysing strips separately would be appropriate.

### Local background estimation and subtraction

It is interesting to note the consistency of the estimated background for individual beads, which is observed within and between arrays. The background estimation used by Illumina takes an average of the five dimmest pixels within a comparatively large area surrounding each bead. This gives a very low estimate for background that is related to the optical properties of the array surface rather than being specific to the sequence attached to each bead. In contrast, background estimation for two-colour arrays typically uses the mean or median value of pixels surrounding each feature, producing higher, more variable estimates. The approach Illumina uses is more akin to a morphological background estimation, which has been shown to perform well for two-colour arrays [6,7].

In other data sets, we have used the predictability of the background signal as a simple diagnostic to identify poor quality arrays on which the background level was considerably higher and more variable than usual (data not shown). When analysing this experiment, we found that subtracting this low estimate of the background was beneficial for detecting differentially expressed genes. At low spike concentrations (around 1 pM), the observed values for the spikes are close to the negative controls. Therefore, when comparing arrays with low spike concentrations that have not been background subtracted, the calculated log-ratios will be biased towards zero as the difference in spike concentration is obscured by the background noise. Background subtraction reduces this bias, although, as anticipated, we see an increase in variability after a log_2 _transformation of the subtracted data. The results of the simplest method of subtracting the background estimates are comparable to those of the model-based approach of normexp. This is due to the low percentage (less than 1%) of negative intensities produced using the subtract method, hence methods that avoid these negative values have little scope for improvement. This is encouraging for users without access to raw data who perform pre-processing using Illumina's default settings.

### Summarisation

Illumina's default summary method was able to handle around 20% of outlier beads before the estimates became noticably biased in our simulations. This provides a rough guideline on how much of an array can be corrupted before the analyst needs to worry about biases creeping into the estimates and inflating the variances. In addition, Illumina's method is better at accommodating asymmetric outliers than regular trimmed means. This is desirable, as these artefacts arise frequently in data sets we have analysed.

### Normalisation

In this study we did not conduct a thorough investigation into normalisation methods. Given the low variability of replicate observations, it is important that the data are not "over-normalised", thus removing potentially interesting biological information. An important conclusion from the spike-in experiment is that the background normalisation recommended by Illumina is not appropriate for some DE analyses. This method is seen to introduce substantial variability into the data, particularly at low intensities, and also to increase the numbers of false positives. Another consequence of this normalisation is that low expression values become negative and cannot be log_2 _transformed. In the spike-in experiment, we found that around 40% of the data were missing on average per array. Illumina keep the bead summary data on the unlogged scale and their model for differential expression takes the relationship between the mean and variance of each bead type into account [14]. DE analyses performed outside of BeadStudio, such as limma *eBayes *[12], *SAM *[15], or other methods, usually require data that have been subjected to a log_2_, or similar, transformation to ensure the gene-wise variances are comparable. Therefore we recommend that only non-normalised data are exported from BeadStudio if they are to be analysed using established statistical methods. Otherwise, a small offset could be added to the intensities to ensure positivity of the background normalised data.

### DE analysis

We find that using the bead type variances as inverse weights increases the evidence for DE for the spikes for each background correction method in most contrasts. At the same time, the log-odds scores for the non-spikes are not affected; this represents a gain in statistical power. The weights also produce more comparable log-odds between processing methods. Less precise observations arising from arrays with quality issues, or intensity-dependent trends in variablity introduced by the chosen pre-processing option, are down-weighted in the analysis. At very low concentrations (less than 1 pM), this improvement was reversed, with DE statistics decreasing for the spikes. Although this would seem undesirable, it indicates that after considering the underlying variability of the observations, it is difficult to distinguish between very small changes in concentration, which is a limitation of any microarray technology. Having access to the bead level data allows bead type variances to be calculated on the appropriate scale so that they may be used in the linear model.

### Annotation

Probe annotation should be given careful consideration during the analysis. The second strip should assist in the investigation of rare transcripts, but it also contains many probes that will not produce any meaningful signal in many gene expression studies. One way of exporting data from BeadStudio is to average the probes for the same gene. We would discourage combining the signal from two probes, as there may be differences in the reliability of the probes, particularly if one maps to an intron or intergenic region. We found the intensities of probes on an array to be related to base composition. In particular, probes with a higher GC content were seen to have a higher intensity, as were probes with a G or C at the first base. These effects were observed on normalised data from strip 1 and persisted in the between-array comparisons. Inflated differential expression statistics were found for probes with 17 to 22 GCs in their sequence.

A possible probe effect is also suggested by the intensity differences in both spikes and negative controls. Given these observations and previous work for Affymetrix arrays, it would seem that more sophisticated methods than background normalisation are needed to account for sequence-specific hybridisation effects. The correlation of observed intensities with the melting temperature or free energy is seen to decline with the spike concentration, presumably as the signal-to-noise ratio diminishes. Similar correlations were seen in Affymetrix spike-in experiments. While it is clear that some of the behaviour of the negative controls can be explained by their thermodynamic properties, 2 – 3% of the variation is explained at best. Since the intensities of the negative controls are reproducible across strips and arrays, it seems implausible that the remainder could consist of random noise. Thus, we conclude that other sources of variation are to be identified.

### Application to other Illumina technologies

In this paper, we describe the advantages of analysing a gene expression experiment using bead level data. We also anticipate that the analysis of other Illumina assays (e.g. GoldenGate, Infinium, DASL) can benefit from using bead level data. For instance, recent genotyping methods for Affymetrix technology successfully use the full raw data and therefore having access to the bead level data is likely to be useful in developing similar methods for Illumina. If log-ratios are required for genotyping, the situation is similar to expression data where the values output by BeadStudio are not on the desired scale. With the raw data, it is possible to obtain log ratios for every bead and then calculate an average and variance for each bead type.

## Conclusion

The main findings presented in this paper are:

• Access to bead level data promotes more detailed quality assessment and more flexible analyses. Bead level data can be summarised on a relevant scale. We were able to use the means and variances of the log_2 _data in the DE analysis to improve our ability to detect known changes in the spikes.

• The background correction and summarisation methods used in BeadStudio reduce bias and produce robust gene expression measurements. However, we find that background normalisation introduces a significant number of negative values and much increased variability.

• Base composition of probes has an effect on intensity and further investigation is required to remove this effect.

## Methods

The raw data were read using the *beadarray *package (version 1.6.0), available through the Bioconductor project [16].

### Image analysis

The foreground estimation algorithm used by Illumina is a two-step process described in more detail in [2]. The main steps in Illumina's image analysis are

i) All pixel intensities are altered using a sharpening transformation. The intensity of a particular pixel is made higher/lower if its intensity is higher/lower in comparison to the intensities of the pixels surrounding it.

ii) Foreground intensities are calculated as a weighted average of signals obtained using the four pixels nearest to each bead centre as a "virtual bead centre". Sharpened pixel intensities are used in the calculation. The value returned is unlogged.

Background intensities are estimated using an average of the five dimmest pixels (unsharpened intensities) within the 17 × 17 pixel area around each bead centre.

We also read the TIFF images using the *EBImage *package [17]. We then created an incidence matrix indicating whether each pixel was within a bead or part of the background. This allowed us to assess the intensity of pixels in the area immediately surrounding each bead. These pixels were ranked in order of increasing intensity to establish the variation in the pixels used to estimate the local background.

### Background correction

The following methods were used to adjust the foreground (*y*_*f*_) of each bead using the corresponding background estimate (*y*_*b*_) to obtain an estimate of the true signal due to hybridisation (*y*).

• No adjustment – Use the estimated foreground (*y *= *y*_*f*_) in the analysis (assume *y*_*b *_= 0).

• Subtract – The estimated background is subtracted from the foreground for each bead (*y *= *y*_*f *_- *y*_*b*_). No guarantee is given against negative values appearing.

• Normexp – A normal-exponential convolution model is fitted to the background subtracted signal (*y *= *y*_*f *_- *y*_*b*_) to adjust the intensities from each strip separately (see [7] for details). This model has the advantage of returning strictly positive intensities.

The background correction method used in BeadScan is the Subtract method.

### Summarisation

Most analyses in this paper used the background adjusted (see above), log_2 _transformed data from replicate beads on a given array and summarised these values using Illumina's default method. This method removes outliers greater than 3 median absolute deviations (MADs) from the median and calculates a mean, standard error and number of observations for the remaining intensities. To look at how robust Illumina's method is relative to other summarisation methods (mean, trimmed mean removing 10% of highest and lowest intensities or median), we measured the bias for each method from simulated data, where varying numbers of outliers were added (from 0% to 40% in increments of 5%). The true values were assumed to be the means calculated from the original data. Data from a good quality BeadChip from this experiment was replaced at random by intensities at the saturation level (2^16^). Saturation artefacts are fairly common in experiments we have analysed, and often occur at the edges of an array. By varying the number of outliers, we can roughly assess the break-down point of Illumina's summary method. Data for each simulation was summarised on the original and log_2 _scale. Bias was computed by subtracting the summary values obtained from the simulated BeadChip from the summary values obtained from the original data for each probe on each array. Per array, per probe variances were also calculated within each simulated data set. The bias values and variances were averaged across arrays and probes within each simulation to produce the values plotted in Figure 2.

### Visualisation of data

We use MA-plots to show the variablity between arrays for different methods. For two given arrays (*k*1 and *k*2) the summarised intensities for a given bead type, *y*_*k*1 _and *y*_*k*2_, were used to calculate log-ratios *M*, where *M *= log_2_(*y*_*k*1_) - log_2_(*y*_*k*2_) and average log intensities *A*, where $A=\frac{1}{2}[\log_{2}(y_{k1})+\log_{2}(y_{k2})]$. The *M*- and *A*-values were then plotted on the y- and x-axis respectively. A value of *M *= 0 indicates no differential expression between arrays, and most probes in the spike-in experiment should be centred around this value.

### Normalisation

The log_2 _summarised data were quantile normalised as in [4]. This approach is reasonable given that the majority of genes do not change between arrays, and hence the intensity distribution between arrays should be the same. In principle, this could affect the intensity of high intensity spikes, although we found the effect to be small. Background normalisation was carried out on the non-normalised, background subtracted data by subtracting the average value of the negative controls on each array output by BeadStudio from the summarised intensities of the non-control probes. *M*- and *A*-values were calculated to allow comparison with quantile normalised results.

### Linear models and contrasts

The limma package [12] was used to assess DE. Bead level data were created using different background correction methods and then summarised to give an expression matrix **Y**, where *y*_*jk *_represents the log_2 _normalised expression of probe *j *on array *k*. A linear model *E*[**y**_*j*_] = **X*α***_*j *_was fitted to each probe, where $y_{j}^{T}$ is the *j*th row of **Y**, and ***α***_*j *_is a vector of coefficients to be estimated for each probe at the 12 different concentrations. The contrasts of interest are given by ***β***_*j *_= **C**^*T*^***α***_*j*_, where **C **is a contrast matrix created to make all pairwise comparisons between concentrations (e.g. 1000 pM vs 300 pM, 300 pM vs 100 pM etc). After empirical Bayes variance shrinkage, the moderated *t*-statistics and log-odds scores for each contrast were analysed separately to assess the performance of different background correction methods. A second series of linear models was fitted to take the variability of bead types into account. We now assume that $var(y_{jk})=\sigma_{j}^{2}/w_{jk}$ where *w*_*jk *_is a weighting factor for bead type *j *on array *k*. Weights $w_{jk}=1/s_{jk}^{2}$, where $s_{jk}^{2}$ is the sample variance calculated using the standard error and the number of observations of bead type *j *on array *k*, were used. Using inverse variances as weights gives less influence to observations with higher variability in the linear model. The coefficients, ***α***_*j*_, were estimated using weighted least squares and contrasts, ***β***_*j*_, were calculated as before.

See supplementary materials for the R code used to fit the linear models.

### Annotation

Probe sequences were BLASTed and BLATed against the corresponding mouse genome and transcriptome, which included UCSC Genome Browser [18], RefSeq, and GenBank transcripts. The subsequent annotation and probe classification were performed with a Perl script, comprising BioPerl modules [19], and relied on transcriptomic annotation tables downloaded from the UCSC Genome Browser. The resulting annotation table is available in supplementary materials.

We defined **A**, **C**, **G **and **T **to be matrices of binary values with *j *= 1,..., ~48,000 rows and *p *= 1, ..., 50 columns to represent the sequence of each probe, where *A*_*jp *_= 1 if the sequence for probe *j *contained an "A" at position *p*, or 0 otherwise.

The total number of As (*a*_*j*_) in the sequence of the *j*'th probe is simply $a_{j}=\sum_{p=1}^{50}a_{jp}$. The total number of Cs (*c*_*j*_) Gs (*g*_*j*_) and Ts (*t*_*j*_) were defined in a similar fashion. The GC content for probe *j *was then defined as *g*_*j *_+ *c*_*j*_,

We then plotted the normalised intensities of the strip 1 probes on a given array in terms of their *a*_*j*_, *c*_*j*_, *g*_*j*_, *t*_*j *_and GC content. Similarly, for a particular contrast, we ranked the same probes according to their log-odds scores and plotted probes with the same GC content together.

The linear model *E*[**y**_*k*_] = **A*α***_*k *_+ **C*β***_*k *_+ **G*γ***_*k*_, was fitted to the intensities and variances of the *k*'th array to estimate coefficients ***α***, ***β***, ***γ ***representing the effect of having an A, C or G at each position, relative to having a T at that position.

The melting temperature, free energy (Δ*G*), entropy (Δ*S*) and enthalpy (Δ*H*) were calculated for each spike and negative control probe using values taken from [20] with code calibrated against OligoCalc [21], although we recognise that these values may not be strictly applicable to 50-mer oligos.

## Authors' contributions

MJD analysed the spike-in experiment and drafted the paper. NLBM performed the re-annotation and helped draft the manuscript. AGL supervised the probe sequence modelling, performed some of the probe thermodynamics analysis and helped draft the manuscript. ST instigated and guided the research project and proof-read the manuscript. MER supervised the analysis, figure preparation and finalised the manuscript. All authors read and approved the manuscript.
